# The Association of the Long Prostate Cancer Expressed PDE4D Transcripts to Poor Patient Outcome Depends on the Tumour's TMPRSS2-ERG Fusion Status

**DOI:** 10.1155/2019/8107807

**Published:** 2019-06-02

**Authors:** Dianne van Strijp, Christiane de Witz, Birthe Heitkötter, Sebastian Huss, Martin Bögemann, George S. Baillie, Miles D. Houslay, Chris Bangma, Axel Semjonow, Ralf Hoffmann

**Affiliations:** ^1^Philips Research Europe, 5656AE Eindhoven, Netherlands; ^2^Gerhard-Domagk-Institute of Pathology, University Hospital Münster, 48149 Münster, Germany; ^3^Prostate Center, University Hospital Münster, 48149 Münster, Germany; ^4^Institute of Cardiovascular and Medical Science, University of Glasgow, G12 8TA Glasgow, Scotland, UK; ^5^Institute of Pharmaceutical Science, King's College London, 150 Stamford Street, London, WC2R 2LS London, UK; ^6^Mironid Ltd, BioCity Scotland, ML1 5UH Newhouse, Scotland, UK; ^7^Department of Urology, 3000CA Erasmus Medical Center, Rotterdam, Netherlands

## Abstract

**Objectives:**

To investigate the added value of assessing transcripts for the long cAMP phosphodiesterase-4D (PDE4D) isoforms, PDE4D5 and PDE4D9, regarding the prognostic power of the ‘CAPRA & PDE4D7' combination risk model to predict longitudinal postsurgical biological outcomes in prostate cancer.

**Patients and Methods:**

RNA was extracted from both biopsy punches of resected tumours (606 patients; RP cohort) and diagnostic needle biopsies (168 patients; DB cohort). RT-qPCR was performed in order to determine PDE4D5, PDE4D7, and PDE4D9 transcript scores in both study cohorts. By RNA sequencing, we determined the TMPRSS2-ERG fusion status of each tumour sample in the RP cohort. Kaplan-Meier survival analyses were then applied to correlate the PDE4D5, PDE4D7 and PDE4D9 scores with postsurgical patient outcomes. Logistic regression was then used to combine the clinical CAPRA score with PDE4D5, PDE4D7, and PDE4D9 scores in order to build a ‘CAPRA & PDE4D5/7/9' regression model. ROC and decision curve analysis was used to estimate the net benefit of the ‘CAPRA & PDE4D5/7/9' risk model.

**Results:**

Kaplan-Meier survival analysis, on the RP cohort, revealed a significant association of the PDE4D7 score with postsurgical biochemical recurrence (BCR) in the presence of the TMPRSS2-ERG gene rearrangement (logrank p<0.0001), compared to the absence of this gene fusion event (logrank p=0.08). In contrast, the PDE4D5 score was only significantly associated with BCR in TMPRSS2-ERG fusion negative tumours (logrank p<0.0001 vs. logrank p=0.4 for TMPRSS2-ERG+ tumours). This was similar for the PDE4D9 score although less pronounced compared to that of the PDE4D5 score (TMPRSS2ERG- logrank p<0.0001 vs. TMPRSS2ERG+ logrank p<0.005). In order to predict BCR after primary treatment, we undertook ROC analysis of the logistic regression combination model of the CAPRA score with the PDE4D5, PDE4D7, and PDE4D9 scores. For the DB cohort, this demonstrated significant differences in the AUC between the CAPRA and the PDE4D5/7/9 regression model vs. the CAPRA and PDE4D7 risk model (AUC 0.87 vs. 0.82; p=0.049) vs. the CAPRA score alone (AUC 0.87 vs. 0.77; p=0.005). The CAPRA and PDE4D5/7/9 risk model stratified 19.2% patients of the DB cohort to either ‘no risk of biochemical relapse' (NPV 100%) or the ‘start of any secondary treatment (NPV 100%)', over a follow-up period of up to 15 years. Decision curve analysis presented a clear, net benefit for the use of the novel CAPRA & PDE4D5/7/9 risk model compared to the clinical CAPRA score alone or the CAPRA and PDE4D7 model across all decision thresholds.

**Conclusion:**

Association of the long PDE4D5, PDE4D7, and PDE4D9 transcript scores to prostate cancer patient outcome, after primary intervention, varies in opposite directions depending on the TMPRSS2-ERG genomic fusion background of the tumour. Adding transcript scores for the long PDE4D isoforms, PDE4D5 and PDE4D9, to our previously presented combination risk model of the combined ‘CAPRA & PDE4D7' score, in order to generate the CAPRA and PDE4D5/7/9 score, significantly improves the prognostic power of the model in predicting postsurgical biological outcomes in prostate cancer patients.

## 1. Introduction

Prostate cancer is the most common nonhematology tumour diagnosed in men in western countries [1, 2]. The natural history of prostate tumours is heterogeneous with, in general, indolent characteristics. However, it includes some forms of the disease that can develop into deadly cancers. Disease-specific mortality is, in particular, small for low-risk cancers [3, 4]. This has led to a paradigm change in the management of men with low-risk profiles, as there is a considerable chance that definitive treatment is not beneficial for these patients but comes with the burden of adverse effects of the primary treatment. Nevertheless, some patients with clinically low-risk characteristics progress after initial intervention [5–7] while others, with more advanced pathological features will experience stable disease during periods of follow-up [8, 9]. This continues to pose the challenge of selecting the most optimal management strategy for each individual patient. While various national guidelines recommend considering conservative management (i.e., active surveillance: AS) of low-risk patients, it remains crucial to select the most suitable patients for this regime, as discontinuation from AS, and switching to active treatment, due to signs of progressive disease, is common in these patient cohorts [10, 11]. Consequently, more advanced protocols for inclusion/exclusion of men to conservative management, monitoring strategies while in AS and measures to switch to definitive treatment are required for optimal prostate cancer patient care [12].

Signalling through the ubiquitous second messenger, cyclic AMP (cAMP) critically impacts the functioning of all cell types in the body. Such actions are mediated through specific effector proteins, namely, protein kinase A (PKA) and Exchange Protein Activated by cAMP (Epac) [14, 15]. These species are sequestered to distinct signalling complexes within cells, conferring a spatial aspect that leads to compartmentalization of signalling. The sole means of degrading cAMP, so as to terminate cAMP signalling, is through the action of cAMP phosphodiesterases (PDEs). In this, the 20+ isoforms encoded by the four gene PDE4 family (PDE4A/4B/4C/4D) play a critical role in the compartmentalized degradation of cAMP, as their isoform-specific N-terminal regions contain motifs that allow for their targeting to distinct signalling complexes [16]. PDE4D5, PDE4D7, and PDE4D9 are a so-called long isoform as each contains both the UCR1 and the UCR2 regulatory domains that allow for regulation by various protein kinases, including PKA and MK2 as well as determining the functional outcome of catalytic unit phosphorylation by ERK [16]. Functionally, these enzymes contribute to the cellular desensitization system towards cAMP and provide nodes that enable cross-talk between signalling pathways involving ERK, p38MAPK, and AMPK [16].

Previously, we described the positive association of PDE4D7 expression and the prostate-specific gene arrangement between the androgen regulated transmembrane protease TMPRSS2 and the ETS transcription factor family member ERG [17]. A study in 2005 demonstrated that the chromosome 21 genomic fusion event TMPRSS2-ERG, between the transmembrane protease serine 2 TMPRSS2 and the members of the ETS (erythroblast transformation-specific) transcription factor family ERG, is common in prostate cancer [13]. The overexpression of ERG, in a majority of prostate cancers, is driven by this fusion event, which switches ERG to fall under the control of the androgen-driven promotor of the TMPRSS2 gene. In a recent whole genome sequencing study, the TMPRSS2-ERG fusion was identified as an early event in the development of prostate cancer [18]. However, while numerous studies have been performed, since its discovery, the role of the TMPRSS2-ERG fusion, in prostate cancer development and progression, is not yet fully understood [19].

Interestingly, we did not find the same positive association for other prostate cancer expressed long PDE4D isoform transcripts, namely, PDE4D5 and PDE4D9. In addition, we reported that the expression of PDE4D7 is inversely correlated to risk of biochemical relapse after prostate cancer surgery and, independently, adds to clinical variables and risk scores like either CAPRA or CAPRA-S [20, 21]. The CAPRA (Cancer of the Prostate Risk Assessment) and CAPRA-S scores are risk models that combine either presurgical (CAPRA) or postsurgical (CAPRA-S) score data relating to routinely available clinical variables (PSA, Gleason score, etc.). These models were shown to have superior power to predict postsurgical patient outcome, either before (CAPRA) or after (CAPRA-S) prostate operation, compared to using the respective input variables alone [22].

Here, we set out to investigate whether we could identify any difference in prediction of postsurgical progression risk by PDE4D7 expression in positive vs. negative TMPRSS2-ERG fusion prostate tumours and, whether, either PDE4D5 or PDE4D9 transcript analysis might similarly contribute to the progression risk, as has been shown for PDE4D7 transcript analysis.

## 2. Patients and Methods

### 2.1. Patient Cohorts and Samples

#### 2.1.1. RP (Radical Prostatectomy) Patient Cohort (n=606)

Patients consecutively managed at a single, large-volume prostate cancer center were included in the study (Martini Klinik, Hamburg, Germany). Two small biopsy punches (~1x2 mm), of a representative resected tumour area, of patients operated on between 2000 and 2004, were collected from the tumours index lesion.

#### 2.1.2. RP*∗* (Radical Prostatectomy*∗*) Patient Cohort (n=130)

Detailed characteristics of this cohort and analysis of the respective gene expression data have been described previously [23].

#### 2.1.3. DB (Diagnostic Biopsy) Patient Cohort (n=168)

From the tumour positive diagnostic biopsy, with the highest Gleason grade per patient, a single biopsy punch (~1x2 mm) was collected. Patients reflected those diagnosed with prostate cancer and having undergone RP between 1994 and 2011 at the Prostate Center (University Hospital Münster, Germany). The local Institutional Review Boards approved the collection of patient tissue for clinical research, with appropriate patient consent (for cohort design see Supplementary Figure 1). Characteristics of this cohort have been published previously [19].

### 2.2. Laboratory Methods

To account for potential tumour heterogeneity, the two tissue punches of the RP cohort were combined before nucleic acid extraction. A potential difference in tumour cellularity of the tissue punches was addressed by normalization of the qPCR results of the PDE4D transcript to four reference genes, which were selected based on stable gene expression across multiple tumour sample types [20]. All used molecular laboratory methods including oligonucleotide primers and probes for RT-qPCR (reverse-transcriptase-quantitative PCR), RNA extraction, and quality control and procedures are to include/discard samples from the statistical analysis as described before by us [20].

### 2.3. RNA Sequencing

#### 2.3.1. RNA Sample Processing

100 ng of total RNA was used as input to remove ribosomal RNA, using Ribo-Zero Gold (Human/Mouse/Rat) rRNA Removal Kit (Illumina Inc.), according to the manufacturer's instructions. For library construction, we used the total of the depleted RNA as input into the Scriptseq V2 RNA-Seq Library Preparation Kit (Epicentre/Illumina Inc.). Prepared RNAseq libraries were sequenced using NextSeq 500 sequencing system (sequencing was done by paired-end at 2 x 75 bp read length providing approx. 80 million total reads per sample).

#### 2.3.2. RNAseq Data Processing

The RNAseq raw data was preprocessed using Illumina bcl2fastq software incorporating filtering by phred scores, thereby reducing low quality reads. Since FFPE degenerates the bases, the sequencing results have been filtered using a scoring algorithm to select reads representing the high-quality fraction. The final score was calculated for a set of reads in a sample as follows. Firstly, the set of reads was aligned against a human reference genome. Then the alignment result, for each read (i.e., the number of bases mapping correctly to the reference genome), was counted per read. The total number of successfully mapped bases was then summed over all reads of the set. This sum was divided by the total number of bases of the set. The resulting relative number is called the EQ score. A score filter selects the subset of reads that contributes to the EQ Score by virtue of a good alignment result (all or most of the bases map correctly to the genome). The derived subset of high-quality reads was then selected for further processing. If reads are mapped by fragmenting them, which may be required when aligning RNA, the measure was calculated based on the fragments alignment quality and the fragments selected accordingly.

#### 2.3.3. Read Quality Filtering

To retain only high-quality reads, the following filtering steps were applied: reads were discarded when >50% of the bases had a phred score below 11; bases at the read ends were removed if the phred score fell below 11; sequencing reads <63 bases and reads with unknown (N) base calls were discarded and sequencing read pairs were kept only if both reads passed the above described quality filter.

#### 2.3.4. Gene Expression Calculation

To ensure comparability of expression values between samples all read counts were normalized by the transcripts per million method (TPM) as implemented in the RSEM algorithm [24].

### 2.4. Data Analysis and Statistics

After quality control of the RNAseq, and the qPCR data, 536 patient samples for the RP cohort and 151 patient samples for the DB cohort were defined eligible for statistical analysis.

Generation of normalized PDE4D transcript expression was performed by subtracting the RT-qPCR Cq of the respective PDE4D transcript from the averaged RT-qPCR Cq of the reference genes. Normalized PDE4D5, PDE4D7, and PDE4D9 expression was transformed to the PDE4D5, PDE4D7, and PDE4D9 scores [20]. Note that we did not use the ΔΔCt method which is used to compare the n-fold expression difference of a gene of interest between two patient cohorts (e.g., treated vs. control) as we aimed to present a score for potential future diagnostic use without the need of a control group. In correlation analysis for various available biological and treatment related outcomes (Table 1) the PDE4D transcript scores were either used as a continuous, or as a categorical variable, defined as (a) PDE4D5/7/9 score (1≤2); (b) PDE4D5/7/9 score (>2 and ≤3); (c) PDE4D5/7/9 score (>3 and ≤4); (d) PDE4D5/7/9 score (>4 and ≤5). The CAPRA risk score and corresponding low (1), intermediate (2), and high-risk (3) categories were calculated as described earlier [22]. Uni- and multivariate Cox regression and Kaplan-Meier analyses were applied to correlate biochemical recurrence (BCR) progression free survival, or secondary treatment (salvage radiation and or androgen deprivation) free survival (STFS) to the PDE4D7 score in the RP cohorts (n=536), and Taylor et al. [23]; n=130) and the DB cohort (n=151). To determine the TMPRSS2-ERG status of patient samples in Exon Array cohorts, we used relative ERG expression values and applied Partitioning Around Medoids (PAM, R-package ‘cluster', k = 2) to assign the patient samples to the ERG positive or negative group based on expression. Decision curve analyses were performed as described [25]. For statistical analysis the software package MedCalc (MedCalc Software BVBA, Ostend, Belgium) was used. The data analysis strategy is outlined in Supplementary Figure 2.

## 3. Results

### 3.1. Association of PDE4D Transcript Scores to Longitudinal Clinical Outcomes Depends on the TMPRSS2-ERG Fusion Status

Firstly, we set out to do Kaplan-Meier survival analysis of the PDE4D7 score categories in TMPRSS2-ERG rearrangement positive vs. gene fusion negative patient samples. In total, we included 536 patient samples with data on TMPRSS2-ERG status, of which we defined 280 (52.2%) as fusion positive, while 256 samples (47.8%) were defined to be absent of this prostate-specific gene fusion event. We selected biochemical recurrence (BCR) as a surrogate endpoint for postsurgical disease progression, due to the significant number of events for this outcome in our studied patient cohorts (Table 1).

**Figure 1:**
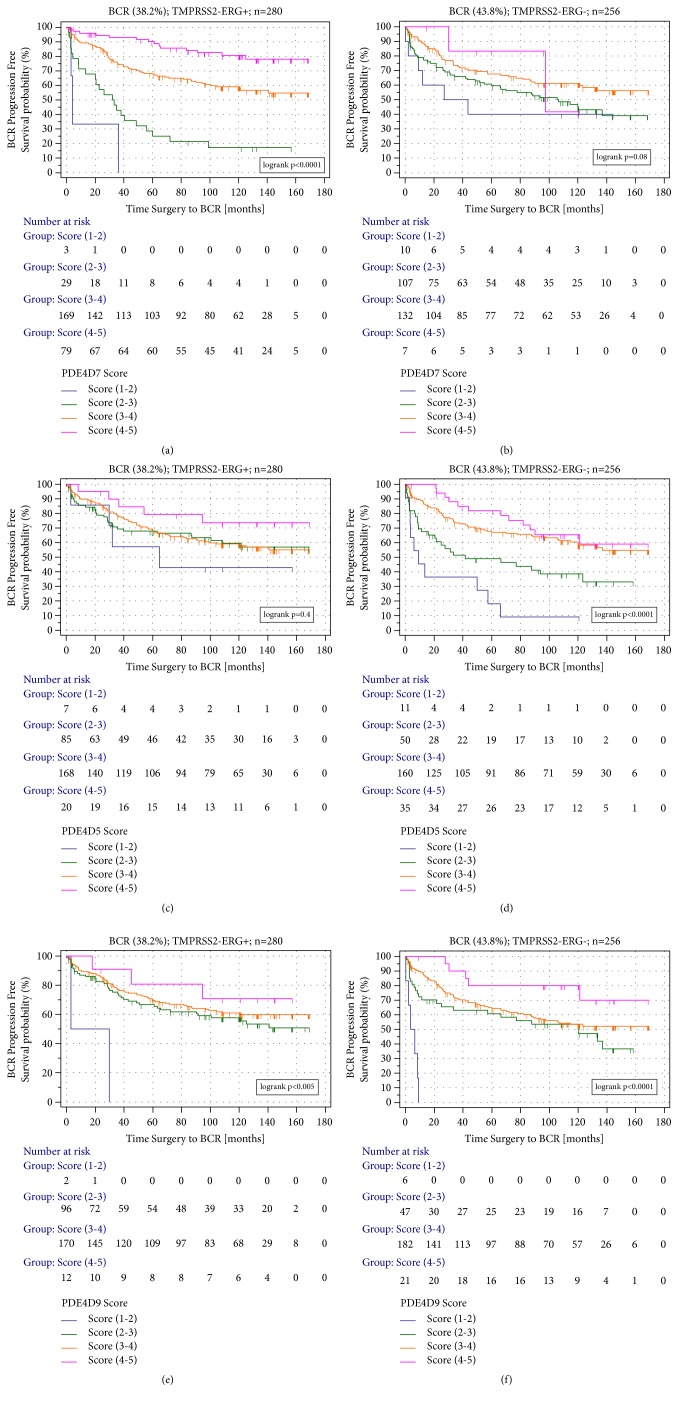
Kaplan-Meier survival analysis of the time to PSA relapse (endpoint BCR: biochemical recurrence) in the RP patient cohort (n=536) for the PDE4D5, PDE4D7, and PDE4D9 scores. (a) Kaplan-Meier analysis of the BCR free survival of the PDE4D7 score in TMPRSS2-ERG positive tumours (n=280). (b) Kaplan-Meier analysis of the BCR free survival of the PDE4D7 score in TMPRSS2-ERG negative tumours (n=256). (c) Kaplan-Meier analysis of the BCR free survival of the PDE4D5 score in TMPRSS2-ERG positive tumours (n=280). (d) Kaplan-Meier analysis of the BCR free survival of the PDE4D5 score in TMPRSS2-ERG negative tumours (n=256). (e) Kaplan-Meier analysis of the BCR free survival of the PDE4D9 score in TMPRSS2-ERG positive tumours (n=280). (f) Kaplan-Meier analysis of the BCR free survival of the PDE4D9 score in TMPRSS2-ERG negative tumours (n=256). Censored patients are indicated by vertical bars. PDE4D5, PDE4D7, and PDE4D9 score categories were defined as PDE4D5/7/9 (1-2): PDE4D5/7/9 scores (1 to <2); PDE4D5/7/9 (2-3): PDE4D5/7/9 scores (2 to <3); PDE4D5/7/9 (3-4): PDE4D5/7/9 scores (3 to <4); PDE4D5/7/9 (4-5): PDE4D5/7/9 scores (4 to <=5).

We observed a clear difference in BCR progression, free survival analysis between the fusion positive vs. negative tumours with a highly significant logrank p (<0.0001) for the PDE4D7 categories in the presence of the rearranged TMPRSS2-ERG gene fusion (Figure 1(a)). The patient group with the highest level of PDE4D7 expression (i.e., PDE4D7 scores 4-5) showed lowest risk of disease progression after surgery in the TMPRSS2-ERG fusion positive cancers. In contrast, in prostate tumours without an ERG gene fusion event, the discrimination in Kaplan-Meier survival between the defined four different PDE4D7 categories was nonsignificant (logrank p = 0.08; Figure 1(b)). Interestingly, when looking at BCR progression free survival analysis of the PDE4D5 score we found the opposite situation compared to what we observed for the PDE4D7 score. Only in gene fusion free tumours did the PDE4D5 score categories significantly (logrank p<0.0001) predict biochemical relapse (Figures 1(c) and 1(d)). We observed a similar result to this for the PDE4D9 score categories with a logrank p<0.0001 in survival analysis in TMPRSS2-ERG negative tumours. However, in contrast to the analysis of PDE4D5 scores, the survival analysis of PDE4D9 score categories in gene fusion positive cancers resulted in a significant association with biochemical recurrence, although with a somewhat weaker p compared to the TMPRSS2-ERG negative tumours (logrank p=0.005 vs. logrank p<0.0001, respectively; Figures 1(e) and 1(f)).

**Figure 2:**
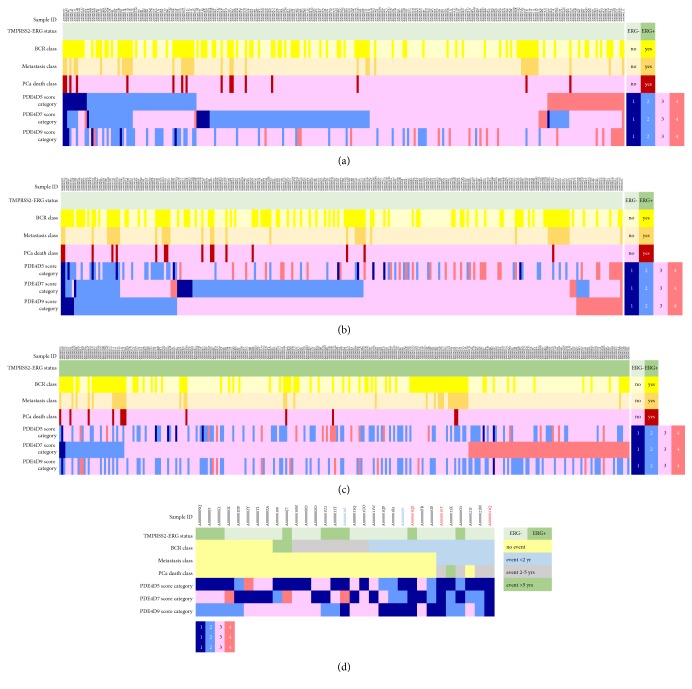
(a) Heatmap of TMPRSS2-ERG negative tumour samples of the RP cohort (n=256); samples are ordered according to their PDE4D5 score from low to high. (b) Heatmap of TMPRSS2-ERG negative tumour samples of the RP cohort (n=256); samples are ordered according to their PDE4D9 score from low to high. (c) Heatmap of TMPRSS2-ERG positive tumour samples of the RP cohort (n=280); samples are ordered according to their PDE4D7 score from low to high. The legends of the graphs and color coding are defined as ‘Sample ID': IDs of the 256 TMPRSS2-ERG negative tumour samples of the RP cohort. ‘TMPRSS2-ERG status': presence (dark green) or absence (light green) of the gene fusion event in a given sample. ‘BCR class': every patient is coded for the presence (dark yellow) or absence (light yellow) of a BCR (biochemical recurrence) event during the >120 months median follow-up. ‘Metastasis class': every patient is coded for the presence (dark orange) or absence (light orange) of a metastases event during the >120 months median follow-up). ‘PCa death class': every patient is coded for the presence (dark red) or absence (light red) of a prostate cancer specific death event during the >120 months median follow-up. PDE4D5, PDE4D7, and PDE4D9 score categories were defined as PDE4D5/7/9 (1-2): PDE4D5/7/9 scores (1 to <2; dark blue); PDE4D5/7/9 (2-3): PDE4D5/7/9 scores (2 to <3; light blue); PDE4D5/7/9 (3-4): PDE4D5/7/9 scores (3 to <4; light pink); PDE4D5/7/9 (4-5): PDE4D5/7/9 scores (4 to <=5; dark pink). (d) RP cohort patient samples (n=31) with the lowest PDE4D5, PDE4D7, and PDE4D9 scores. The legends of the graph and color coding are defined as above with the change of a color coding for BCR, Metastasis, and PCa Death class according to a time interval to the event; light yellow: no event during >120 months median follow-up; light blue: <2 years to the event during >120 months median follow-up; light grey: 2-5 years to the event during >120 months median follow-up; light green: >5 years to the event during >120 months median follow-up.

Next, we investigated to what extent the score categories for the three different prostate cancer expressed PDE4D transcripts were determined to be mutually exclusive in individual patient samples or, whether, the same score category (e.g., [1, 2] or [4, 5]) was seen across the same samples for the three long form PDE4D splice variants we analysed here. For this we plotted a heatmap that included all 536 patient samples, with an initial split between TMPRSS2-ERG gene fusion negative (Figures 2(a) and 2(b)) vs. fusion positive (Figure 2(c)) samples. While the samples within the TMPRSS2-ERG negative samples were ordered according to their PDE4D5 or PDE4D9 score category (Figures 2(a) and 2(b), respectively) the samples that were positive for the gene fusion were found to order according to their PDE4D7 score category from low to high (Figure 2(c)). The heatmaps replicated the results of the Kaplan-Meier survival analysis, with more events in the lower PDE4D isoform score categories (Figures 2(a)–2(c)). However, as can be appreciated, the PDE4D transcript score categories are, to some extent, non-overlapping within a patient sample. When focusing on the lowest score category (i.e., all scores for PDE4D5/4D7/4D9 between score 1 and score 2) we identified 31 samples with at least one of the three PDE4D transcripts with a score category between score 1 and score 2 (Figure 2(d)). For three samples (marked in bold red) we measured the lowest score category for all three of these long form PDE4D transcripts while for two samples (marked in bold blue) at least two PDE4D transcripts belonged to the lowest score category. For the other 26 samples only one PDE4D splice variant was expressed at very low levels (i.e., score between 1 and 2), while the two other isoforms showed higher expression levels in these samples. The risk, of either developing metastases or dying from prostate cancer (6 and 5 out of the 31 patients, respectively), increases strongly with reduced expression levels of multiple prostate-expressed long PDE4D isoforms (Figure 2(d)). Also, the time scale after surgery, to an event like BCR, was generally shorter (<2 years) for those patients having at least two low PDE4D transcript scores (between either scores 1-2 and/or scores 2-3). Vice versa, the higher the expression level of at least one of the three PDE4D splice variants, the less likely was the chance of the patient in experiencing BCR after surgery. However, in instances where such an event occurred, it was typically on a longer time scale (2-5 years and, in some cases, >5 years after primary treatment).

Taken together, these data indicate that next to PDE4D7 transcript analyses, analysis of transcript levels of the long PDE4D5 and PDE4D9 isoforms may also have significant prognostic value in prostate cancer. Therefore, we hypothesized that the addition of the PDE4D5 and PDE4D9 scores, to that of the PDE4D7 score, might increase the power to predict postsurgical risk of disease progression, either over various clinical variables or over the previously reported prognostic PDE4D7 model [13, 19].

### 3.2. Logistic Regression Model of Clinical Variables and Prostate Cancer Expressed Long PDE4D Transcripts

To test this concept, we developed a prognostic model to include the clinical CAPRA score [22] together with the transcript scores for the PDE4D5, PDE4D7 and PDE4D9 long isoforms. For model development we used the RP (n=536) and RP*∗* cohorts (n=130). We performed logistic regression analysis to predict postsurgical biochemical relapse in the RP and RP*∗* cohorts in order to estimate the weights for the CAPRA score as well as for the PDE4D transcripts. The coefficients were calculated by logistic regression. Next, we adjusted the initial coefficients, after logistic regression analysis, of the four model inputs on the RP*∗* cohort by calculating an average of the coefficients for the RP and RP*∗* cohorts, thus taking the heterogeneity of different patient groups into account. The final CAPRA & PDE4D5/7/9 model (co1*∗*PDE4D5 score + co2*∗*PDE4D7 score + co3*∗*PDE4D9 score + co4*∗*CAPRA score; Supplementary Table 1) was tested for its prognostic power to predict BCR, as well as start of secondary treatment after surgery (i.e., radiation, or hormone deprivation), in the independent DB patient cohort. For any other outcome, such as either metastases or death, we used the RP and RP*∗* cohorts (note: these clinical endpoints were not used during model development).

### 3.3. Kaplan-Meier Survival Analysis of the CAPRA & PDE4D5/7/9 Model

**Figure 3:**
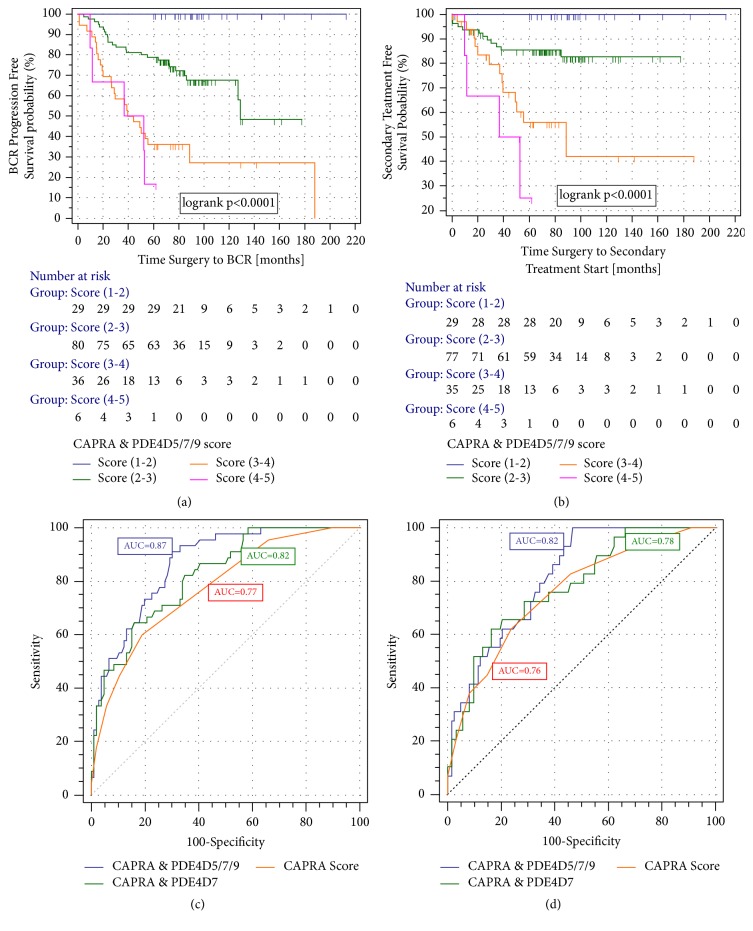
(a) Kaplan-Meier analysis of the biochemical recurrence (BCR) free survival in the diagnostic biopsy patient (DB) of the categorized CAPRA and PDE4D5/7/9 combination score. (b) Kaplan-Meier analysis of the postsurgical of secondary treatment free survival (STFS) time in the diagnostic biopsy patient (DB) cohort of the categorized CAPRA & PDE4D5/7/9 combination score. The CAPRA and PDE4D5/7/9 combination model was developed by logistic regression using data of the RP and RP patient cohort and used as such for testing in the DB patient cohort. The model score was transformed into a CAPRA & PDE4D5/7/9 score distribution between 1 and 5 equivalent to how the individual PDE4D transcript scores were generated [13]. Censored patients are indicated by vertical bars. PDE4D5/7/9 score categories were defined as PDE4D5/7/9 (1-2): PDE4D5/7/9 scores (1 to <2); PDE4D5/7/9 (2-3): PDE4D5/7/9 scores (2 to <3); PDE4D5/7/9 (3-4): PDE4D5/7/9 scores (3 to <4); PDE4D5/7/9 (4-5): PDE4D5/7/9 scores (4 to <=5). (c) ROC curve analysis of 5-year biochemical recurrence in the DB cohort (n=151) of the CAPRA score (orange curve; AUC=0.77) vs. the CAPRA and PDE4D7 (green curve; AUC=0.82) vs. the CAPRA & PDE4D5/7/9 (blue curve; AUC=0.87) logistic regression models. (d) ROC curve analysis of 5-year postsurgical secondary treatment free survival in the DB cohort (n=151) of the CAPRA score (orange curve; AUC=0.76) vs. the CAPRA and PDE4D7 (green curve; AUC=0.78) vs. the CAPRA and PDE4D5/7/9 (blue curve; AUC=0.82) logistic regression models.

In Kaplan-Meier survival analysis the CAPRA & PDE4D5/7/9 model stratified 29 men (19.2%) of the total cohort (n=151) within the lowest score class (between scores 1-2) into a patient group with no risk over the follow-up period of 60 to 200 months of PSA relapse, nor any risk of starting secondary treatments (Figures 3(a) and 3(b)).

By slightly increasing the cut-off of this model score category from (scores 1-2) to (scores 1-2.1), the number of men in this group with no risk of postsurgical disease progression increased from 29 to 36 subjects (23.8%; data not shown). In contrast, the patient with the highest categories of CAPRA & PDE4D5/7/9 scores of (score 3-4 and (score 4-5) experience a risk of biochemical progression within 5 years after surgery of 63.9% and 83.3%, respectively. Similarly, the risk of undergoing secondary treatment was estimated from the survival analysis, within a period of 5 years post-surgery, as 44.1% and 75% for these two patient groups, respectively (Figures 3(a) and 3(b)).

### 3.4. ROC Curve Analysis of the CAPRA & PDE4D5/7/9 Model

For the DB cohort (as above), we iused BCR and start of secondary therapy as clinical outcomes. Thus, we compared the CAPRA & PDE4D5/7/9 model with the, previously presented, CAPRA & PDE4D7 model [21]. For both we tested clinical endpoints, identifying an increase in the AUC (Area Under the Curve) of 10% and 6%, respectively, compared to the CAPRA score alone, and 5% and 4%, respectively, for the CAPRA & PDE4D7 model (Figures 3(c) and 3(d)).

To further explore this, we tested outcomes other than biochemical relapse. As we developed the combination model of the CAPRA and the PDE4D transcript scores, using BCR as an endpoint in the two radical prostatectomy cohorts (RP and RP*∗*), we did not test the model on that endpoint in these cohorts. Instead we used other outcomes for evaluation, namely the progression to metastases after surgery or death from prostate cancer after primary (i.e., RP), or secondary, treatments (i.e., SRT: salvage radiation therapy; SADT: salvage androgen deprivation therapy), to investigate any potential added value of combining PDE4D5 and PDE4D9 transcript scores with our previous CAPRA and PDE4D7 model.  Table 2 provides an overview of the increase in AUC's (areas under the curves) of up to 12%, and up to 12%, comparing the use of either the CAPRA score, the CAPRA and PDE4D7 score model, or the CAPRA and PDE4D5/7/9 score model. These data indicate that the use of additional prostate relevant long form PDE4D transcripts can increase the prognostic power of our previously published combination model of the CAPRA and PDE4D7 score.

### 3.5. Decision Curve Analysis of the CAPRA and PDE4D5/7/9 Model

**Figure 4:**
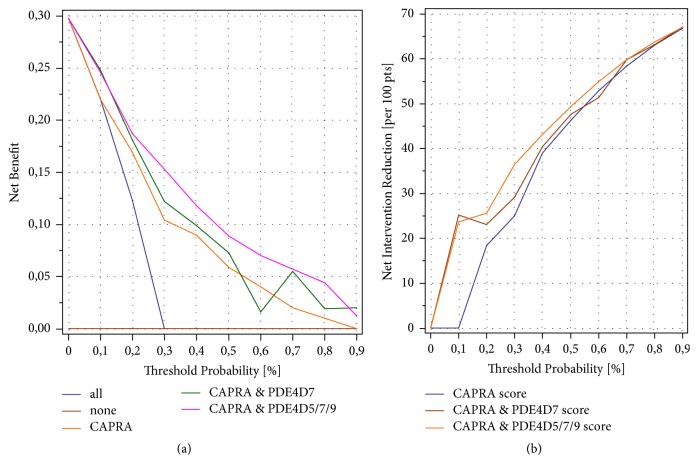
(a) Decision curve analysis in the diagnostic biopsy (DB) patient cohort of the net benefit of five different treatment decision strategies (treat all, treat none, treat based on the CAPRA score, treat based on the CAPRA and PDE4D7 score, treat based on the CAPRA and PDE4D5/7/9 score) for men at risk of disease recurrence within 5 years after surgery. In total 45 of the 151 investigated patients failed the initial primary treatment of surgery by PSA recurrence (29.8%) within 5 years after intervention. Treatment strategies were tested for their net benefit across indicated threshold probabilities (0.05 step size) to trigger prostate surgery based on the probability of future disease recurrence. The CAPRA scores, the CAPRA and PDE4D7 scores, and the CAPRA and PDE4D5/7/9 scores were converted into 5-year BCR probabilities with logistic regression on the BP cohort (n=151 men with completed 5-year follow-up) before estimating net benefit. (b) Net reduction analyses demonstrating in how many patients a resection can be avoided based on the predicted risk of BCR derived from the CAPRA score and the CAPRA and PDE4D7 and CAPRA and PDE4D5/7/9 scores, respectively.

Decision curve analysis is a net benefit analysis that compares the true-positive to the weighted false-positive rates across different risk thresholds that a clinician/patient might want to accept [26]. We explored the net benefit of avoiding primary treatment, based on the predicted risk of a PSA relapse after surgery for the CAPRA score by comparing the utility of the CAPRA and PDE4D7 model versus the CAPRA and PDE4D5/7/9 combination model. Such an analysis demonstrated that both or our models showed better net benefit compared to the “treat all” strategy, while the CAPRA and PDE4D5/7/9 combination model provided the best net benefit across all modeled decision thresholds (Figure 4(a)). Similarly, the net reduction analysis in primary treatment revealed a substantial difference in treatment reduction between using the CAPRA score alone and the CAPRA and PDE4D5/7/9 combination model, across all decision thresholds (Figure 4(b)). Thus, the addition of PDE4D5 and PDE4D9 scores to the CAPRA and PDE4D7 model clearly improves the net benefit in decision curve analysis. Importantly, it provides a potential means of more effectively reducing the number of interventions per 100 patients compared to either the CAPRA model alone or the CAPRA and PDE4D7 combination model.

Thus, here we demonstrate that our previously formulated CAPRA and PDE4D7 risk model can be further improved by adding scores for the long PDE4D5 and PDE4D9 transcripts into the model. The rationale for this added prognostic benefit of PDE4D5 and PDE4D9 is supported by the differences in prediction power between TMPRSS2-ERG positive vs. gene fusion negative patient tumours. Thus, by complementing PDE4D7 with the two other prostate cancer-relevant PDE4D transcripts, namely, PDE4D5 and PDE4D9, we have formulated a more effective prognostic model that has potential for assessing the risk of disease progression before primary intervention in prostate cancer.

## 4. Discussion

We have previously proposed that a predictive model of the clinical risk algorithm CAPRA, in combination with the prostate cancer biomarker PDE4D7, provides value to prostate cancer risk stratification [20, 21]. Although we were able to demonstrate that PDE4D7 transcript analysis adds independent value to the clinical CAPRA model and significantly improves the prognostic power to predict postsurgical disease progression, we set out here to see if there was a way to increase further the value of this CAPRA and PDE4D7 combination model. In our previous work we identified expression differences of various long PDE4D isoforms in primary tumour material that were different for the prostate cancer specific TMRPSS2-ERG gene rearrangement [27]. However, due to limited number of patients and progression events we were not able to investigate whether this phenomenon might also translate into differences of risk prediction subject to the presence or absence of the genomic variation.

Here, however, we have been able to dissect the impact of three different PDE4D transcripts, namely those for the PDE4D5, PDE4D7, and PDE4D9 long isoforms, on the risk of postsurgical disease progression, depending on the genomic background of the patient's tumour. Interestingly, PDE4D7 was found to be associated significantly with posttreatment disease recurrence in a TMRPSS2-ERG fusion positive background, while being of reduced prognostic value in patients without this particular gene fusion event. In contrast PDE4D5 and PDE4D9 transcript levels proved to be highly prognostic in a non-fusion genomic background, while PDE4D9 was less so. And PDE4D5 was not significantly associated with disease progression when the TMRPSS2-ERG genomic fusion event was present.

Lately, multiple genomics studies have identified the* PDE4D* gene as a putative genomic driver/suppressor gene of (prostate) cancer [18, 28, 29]. Indeed, one of these studies even identified differences in the evolution of TMPRSS2-ERG^+^ vs. TMPRSS2-ERG^−^ prostate cancers, based on whole genome sequencing of 112 primary and metastatic prostate tumours [29]. While in ERG^+^ rearranged tumours, the earliest homozygous deletions appeared in region chr5:55-59 Mb in ERG^−^ cancers, losses at chr5:60-100 Mb, covering the well-known affected gene CHD1, were reported. Intriguingly, exon 1, as well as exons 1-3, which specifically encode the isoform-specific N-terminal portions of PDE4D5 and PDE4D7, respectively, is located between the region chr5:59-60 Mb. The differences in genomic rearrangement, as described for chromosome 5, in the different TMPRSS2-ERG fusion background may explain, to some extent, the variability in PDE4D long transcript expression, as described by us earlier [27]. In this study we have exploited and extended this to allow for the PDE4D long isoform-specific prognosis of postsurgical risk of disease progression.

Active surveillance (AS) has become an accepted treatment alternative and is recommended by the national guidelines for men with low- and very-low risk prostate cancer [30]. The guiding principle of AS is to delay, not to avoid, the primary treatment. The switch from AS to active intervention should be taken while the treatment intent is still curative. Consequently, men in AS have to follow strict monitoring schedules as discontinuation and switch to active treatment takes place at the earliest sign of disease progression, such as a rise in PSA, a biopsy Gleason score, or clinical stage migration. Recently, the 10-year outcomes of the ProtecT trial were published [31]. The aim of this randomized controlled trial, which was started in the early 2000 and involving multiple clinical sites across the UK, was to evaluate the effectiveness, cost-effectiveness, and acceptability of treatments (i.e., active monitoring vs. active intervention) for men with localised prostate cancer. Disease-specific mortality was low in all treatment arms of the trial. Moreover, the authors could not conclude, at median follow-up of 10 years, a significant difference in prostate cancer mortality, irrespective of the treatment assigned [31]. Taking the low mortality risk of men in the active monitoring arm of the ProtecT trial into consideration, it is questionable as to what extent the observed changes in clinical presentation of the disease, in an AS setting, correlates with true biological disease progression.

Currently, new technology such as either multi-parametric MRI or genomics, is being considered for stratification of men to AS or for monitoring of men in AS [32, 33]. While the longitudinal cost of AS has been estimated to reach the same order of magnitude as for various primary interventions [34] together with the cost of repeated biopsies, in particular [35], any newly implemented technical tool might only be cost-effective if its use will lead to less men discontinuing AS and facilitating decisions that allow for a switch to definitive treatment and/or significantly reduced surveillance schedules (or even avoided in some patients).

We propose that the combination of a clinical metric, such as the CAPRA score, together with genomic biomarkers such as those presented here, namely evaluation of PDE4D5/7/9 long form transcripts, offer a potentially highly effective means for predicting the future risk of a patient to experience disease progression. Such an approach then may provide future support for selecting patients to be included into modified AS regimens that require, compared to current regimens, a very much reduced requirement for follow-up studies over pre-defined time periods after the start of AS.

## 5. Conclusions

We demonstrate that the prognostic power of analysing the presurgical CAPRA score together with the prostate cancer biomarker PDE4D7 (CAPRA and PDE4D7) can be significantly improved by adding in analyses of transcript level scores for long PDE4D isoforms PDE4D5 and PDE4D9, providing a novel risk model (CAPRA and PDE4D5/7/9). The AUC of the base model of the CAPRA score alone was increased by 10%, from 0.77 to 0.87, when combined with all three prostate cancer relevant long PDE4D transcripts into a single risk prediction algorithm. The resulting risk score is positively correlated with increasing risk of postsurgical disease progression. The patient group with lowest risk score category, as defined here, represents the lowest possible progression risk within the validation cohort with no events occurring during the examined period of follow-up. In contrast, the patient group within the highest risk score category experiences a close to 100% probability of experiencing disease progression after primary therapy.

## 6. Limitations

The retrospective nature of this study provides a potential limitation in the interpretation of the results towards a prospective setting. Furthermore, all study patients were undergoing surgery as a primary treatment. Patient outcomes in terms of the investigated disease progression endpoints might have been influenced by the applied treatment which limits the interpretation towards and active surveillance setting.

## Figures and Tables

**Table 1:** Aggregated summary of the characteristics of the studied patient cohorts. (A) Demographics of the radical prostatectomy (RP) patient cohort including the 536 patients eligible for statistical data analysis. For patient age, preoperative PSA, percentage of tumour in biopsy, prostate volume, and PSA density the min and max values in the cohort are shown; median and IQR (interquartile range) are shown in parentheses. Pre- and postsurgical pathology is given (*Note.* extracapsular extension was derived from pathology stage information). The outcome category illustrates the cumulative 5- and 10-year biochemical recurrence (BCR) and clinical recurrence to metastases (CR) postsurgical primary treatment. The treatment category lists the cumulative 5- and 10-year start to SRT (salvage radiation therapy) or SADT (salvage androgen deprivation therapy) after surgery. Mortality is shown as prostate cancer specific survival (PCSS) as well as overall survival (OS) (N/A=not available). (B) Demographics of the diagnostic biopsy (DB) patient cohort. In total diagnostic needle biopsy tissues of 151 were eligible for statistical data analysis. The demographics and clinical data of this cohort are presented equivalent to the RP cohort (N/A=not available).

	Parameter	(A) RP cohort (n=536)	(B) DB cohort (n=151)
*Demographic & Clinical* Range (median; IQR)	Age range (at RP)	41.3-74.5 (62.5; 7.5)	47.4-77.4 (64.9; 8.5)
	Preoperative PSA range	0.18-120 (7.1; 6.2)	2.0-49.1 (8.1; 5.7)
	Percent tumour in biopsy range	0.2-80.0 (10.6; 20.2)	N/A
	Prostate Volume range	9-244 (41.0; 21.0)	13.6-148.0 (38.5; 19.2)
	PSA density range	0.01-4.0 (0.17; 0.16)	0.03-1.6 (0.2; 0.17)

*CAPRA Risk Category* Number of patients (percentage)	Low Risk (CARPA 0-2)	199 (37.1%)	38 (25.2%)
	Intermediate Risk (CAPRA 3-5)	273 (50.9%)	82(54.3%)
	High Risk (CAPRA>5)	44 (8.2%)	31 (20.5%)
	N/A	20 (3.7%)	-

*Pre-Surgery Pathology* Number of patients (percentage)	Biopsy Gleason 3+3 (GG1)	282 (52.6%)	77 (51.0%)
	Biopsy Gleason 3+4 (GG2)	172 (32.1%)	38 (25.2%)
	Biopsy Gleason 4+3 (GG3)	46 (8.6%)	20 (13.2%)
	Biopsy Gleason >=4+4 (>=GG4)	36 (6.7%)	16 (10.6%)
	cT1	348 (64.9%)	97 (64.2%)
	cT2	175 (32.6%)	
	cT3	13 (2.3%)	54 (35.8%)
	N/A	1 (0.2%)	-

*Post-Surgery Pathology* Number of patients (percentage)	Pathology Gleason 3+3 (GG1)	176 (32.8%)	46 (30.5%)
	Pathology Gleason 3+4 (GG2)	268 (50.0%)	52 (34.4%)
	Pathology Gleason 4+3 (GG3)	69 (12.9%)	31 (20.5%)
	Pathology Gleason >=4+4 (>=GG4)	23 (4.3%)	22 (14.6%)
	pT2	312 (58.2%)	88 (58.3%)
	pT3	224 (41.8%)	63 (41.7%)
	pT4	0 (0%)	0 (0%)
	Positive Surgical Margins	197 (36.8%)	33 (21.9%)
	Extra-Capsular Extension (=T3a)	139 (25.9%)	37/151 (24.5%)
	Seminal Vesicle Invasion	87 (16.2%)	N/A
	Lymph Node Invasion	17 (3.2%)	10 (6.6%)

*Follow-up* (months)	Mean	105.1	73.7
	Median	120.2	73.6

*Outcome*– Number events/total patient number (percentage)	BCR within 5 years	169/480 (35.2%)	45/151 (29.8%)
	BCR within 10 years	210/402 (52.2%)	-
	CR within 5 years	46/472 (9.7%)	4/151 (2.6%)
	CR within 10 years	61/337 (18.1%)	-

*Salvage Treatment* – Number events/total patient number (percentage)	SRT within 5 years	130/475 (27.4%)	12/151 (7.9%)
	SRT within 10 years	164/381 (43.0%)	-
	SADT within 5 years	75/467 (16.1%)	16/151 (10.6%)
	SADT within 10 years	110/350 (31.4%)	-

*Survival*– Number events/total patient number (percentage)	PCSS within 5 years	13/453 (2.9%)	1/151 (0.7%)
	PCSS within 10 years	25/304 (8.2%)	-
	OS within 5 years	25/465 (5.4%)	1/151 (0.7%)
	OS within 10 years	51/331 (15.4%)	-

**Table 2:** Overview of the AUCs for the CAPRA score, the CAPRA and PDE4D7, and the CAPRA and PDE4D5/7/9 regression models to predict multiple endpoints in various patient cohorts. The patient cohort that was used for the respective endpoint is indicated including the number of patients with respective follow-up periods. The tested clinical endpoints are given including the number and percentage of the respectively tested events. *Note.* The CAPRA score is calculated based on [23]; however, as the information on the number of positive biopsy cores was missing for the RP*∗* cohort the CAPRA score for this cohort was calculated using patient age, pre-operative PSA, biopsy Gleason score, and clinical stage only. The influence of the missing information on the biopsy cores was very limited as tested on the RP as well as the DB cohort (data not shown).

Patient Cohort	Tested Clinical Endpoint (post treatment)	# events	CAPRA&PDE4D5/7/9 Score	CAPRA&PDE4D7 Score	CAPRA Score
			AUC		
RP*∗* (n=130)	metastases (post-surgery)	8 (6.2%)	0.86	0.82	0.74

DB (n=151)	5-yr PSA recurrence (post-surgery)	45 (19.8%)	0.87	0.82	0.77

DB (n=151)	5-yr start of secondary treatment (post-surgery)	27 (17.9%)	0.82	0.78	0.76

RP (n=220)	10-yr prostate cancer death (post-surgery) (pGleason >6)	21 (11.1%)	0.78	0.78	0.74

RP (n=86)	10-yr prostate cancer death (post-SRT)	18 (20.9%)	0.78	0.76	0.7

RP (n=61)	10-yr prostate cancer death (post-SADT)	17 (27.9%)	0.74	0.72	0.67

## Data Availability

Previously reported gene expression profiling data were used to support this study and are available at https://www.ncbi.nlm.nih.gov under the accession number GSE21034 and are described in detail previously [Taylor et al., 2010]. These prior studies (and datasets) are cited at relevant places within the text as references [20]. The clinical patient data of the investigational studies (i.e., the RP and the DB patient cohort) used to support the findings of this study have been made available in an aggregated format (Table 1 of the manuscript). In order to protect patient privacy the clinical data of the individual patients have not been made available. The qPCR data generated to support the findings of this study may be released upon request to the corresponding author subject to respective scientific purpose of the use of the data and arrangement of respective data transfer agreements.

## Abbreviations

AS: Active surveillance
RP: Radical prostatectomy
DB: Diagnostic biopsy
BCR: Biochemical recurrence
STFS: Secondary treatment free survival
PCSS: Prostate cancer specific survival
OS: Overall survival
SRT: Salvage radiation therapy
SADT: Salvage androgen deprivation therapy
HR: Hazard ratio
CI: Confidence interval
NPV: Negative predictive value
ROC: Receiver Operating Characteristics
AUC: Area under the curve
DCA: Decision curve analysis
CAPRA: Cancer of the Prostate Risk Assessment.

## Supplementary Materials

Supplementary Materials Supplementary Figure 1. Summary overview of the design of the two patient study cohorts. (A) Design of the radical prostatectomy (RP) cohort. Patients were operated at the Martini Klinik, Hamburg, Germany, between 2000 and 2004. (B) Design of the diagnostic biopsy (DB) cohort. Patients were operated at the University Hospital Muenster, Germany between 1994 and 2011. For clinical characteristics of the patient cohorts, see Table 1 in the main manuscript. Supplementary Figure 2. Analysis design of the generated PDE4D5/7/9 score data in the two patient study cohorts. The surgery cohort (RP) included 606 patients of which 536 were eligible for statistical data analysis after quality control of the RT-qPCR and the RNAseq data and after removal of patients with adjuvant and neo-adjuvant hormone therapy before or after surgery. The RP*∗* cohort (Taylor et al., 2010) consisted of 178 patients of which 130 patients were included into this study. The diagnostic biopsy (DB) cohort comprised 168 patients of which 151 were eligible for statistical data analysis after quality control of the RT-qPCR data. For characteristics of the patient cohorts see Table 1 in the main manuscript. Supplementary Table 1. The CAPRA*∗* and PDE4D579 score combination model was developed by logistic regression of the CAPRA score and the normalized PD4D5, PDE4D7, and PDE4D9 expression values in the RP patient cohort (n=480 with completed 5-years follow-up after surgery) and in the RP*∗* cohort (n=130; Taylor et al., 2010). The logistic regression coefficients for the two cohorts were averaged to generate a mean coefficient for PD4D5, PDE4D7, and PDE4D9 and the CAPRA score based on the data of two independent patients cohorts in order to take the variation of different patient groups into account. These coefficients were used as weights in the final CAPRA and PDE4D579 regression model.* Note. *The CAPRA score is calculated based on Cooperberg and al., 2005; however, as the information on the number of positive biopsy cores was missing for the RP*∗* cohort such that the CAPRA score for this cohort was calculated using only patient age, preoperative PSA, biopsy Gleason score, and clinical stage. The influence of the missing information on the biopsy cores was very limited as tested on the RP as well as the DB cohort (data not shown).
