# Supplementary material for: The Association of the Long Prostate Cancer Expressed PDE4D Transcripts to Poor Patient Outcome Depends on the Tumour's TMPRSS2-ERG Fusion Status
Source: Prostate Cancer. 2019 Jun 2;2019:8107807. doi: 10.1155/2019/8107807 (PMC6582815; doi:10.1155/2019/8107807)
Supplement: Supplementary Materials — Supplementary Figure 1. Summary overview of the design of the two patient study cohorts. (A) Design of the radical prostatectomy (RP) cohort. Patients were operated at the Martini Klinik, Hamburg, Germany, between 2000 and 2004. (B) Design of the diagnostic biopsy (DB) cohort. Patients were operated at the University Hospital Muenster, Germany between 1994 and 2011. For clinical characteristics of the patient cohorts, see Table 1 in the main manuscript. Supplementary Figure 2. Analysis design of the generated PDE4D5/7/9 score data in the two patient study cohorts. The surgery cohort (RP) included 606 patients of which 536 were eligible for statistical data analysis after quality control of the RT-qPCR and the RNAseq data and after removal of patients with adjuvant and neo-adjuvant hormone therapy before or after surgery. The RP∗ cohort (Taylor et al., 2010) consisted of 178 patients of which 130 patients were included into this study. The diagnostic biopsy (DB) cohort comprised 168 patients of which 151 were eligible for statistical data analysis after quality control of the RT-qPCR data. For characteristics of the patient cohorts see Table 1 in the main manuscript. Supplementary Table 1. The CAPRA∗ and PDE4D579 score combination model was developed by logistic regression of the CAPRA score and the normalized PD4D5, PDE4D7, and PDE4D9 expression values in the RP patient cohort (n=480 with completed 5-years follow-up after surgery) and in the RP∗ cohort (n=130; Taylor et al., 2010). The logistic regression coefficients for the two cohorts were averaged to generate a mean coefficient for PD4D5, PDE4D7, and PDE4D9 and the CAPRA score based on the data of two independent patients cohorts in order to take the variation of different patient groups into account. These coefficients were used as weights in the final CAPRA and PDE4D579 regression model. Note. The CAPRA score is calculated based on Cooperberg and al., 2005; however, as the information on the number of positive [file 8107807.f1.docx]

**­­­ Supplementary Figure 1**

**A) B)**


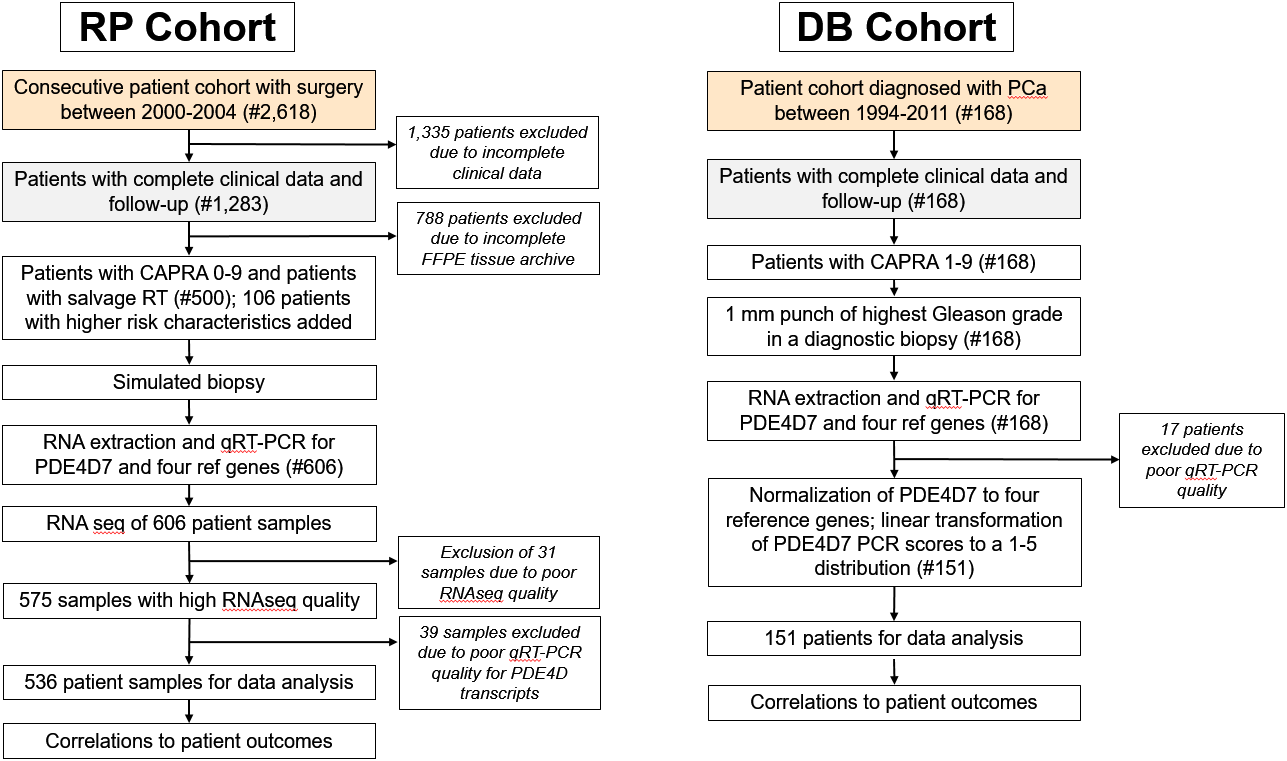


Summary overview of the design of the two patient study cohorts. **(A)** Design of the radical prostatectomy (RP) cohort. Patients were operated at the Martini Klinik, Hamburg, Germany between 2000-2004. **(B)** Design of the diagnostic biopsy (DB) cohort. Patients were operated at the University Hospital Muenster, Germany between 1994-2011. For clinical characteristics of the patient cohorts see Table 1 in the main manuscript.

**Supplementary Figure 2**


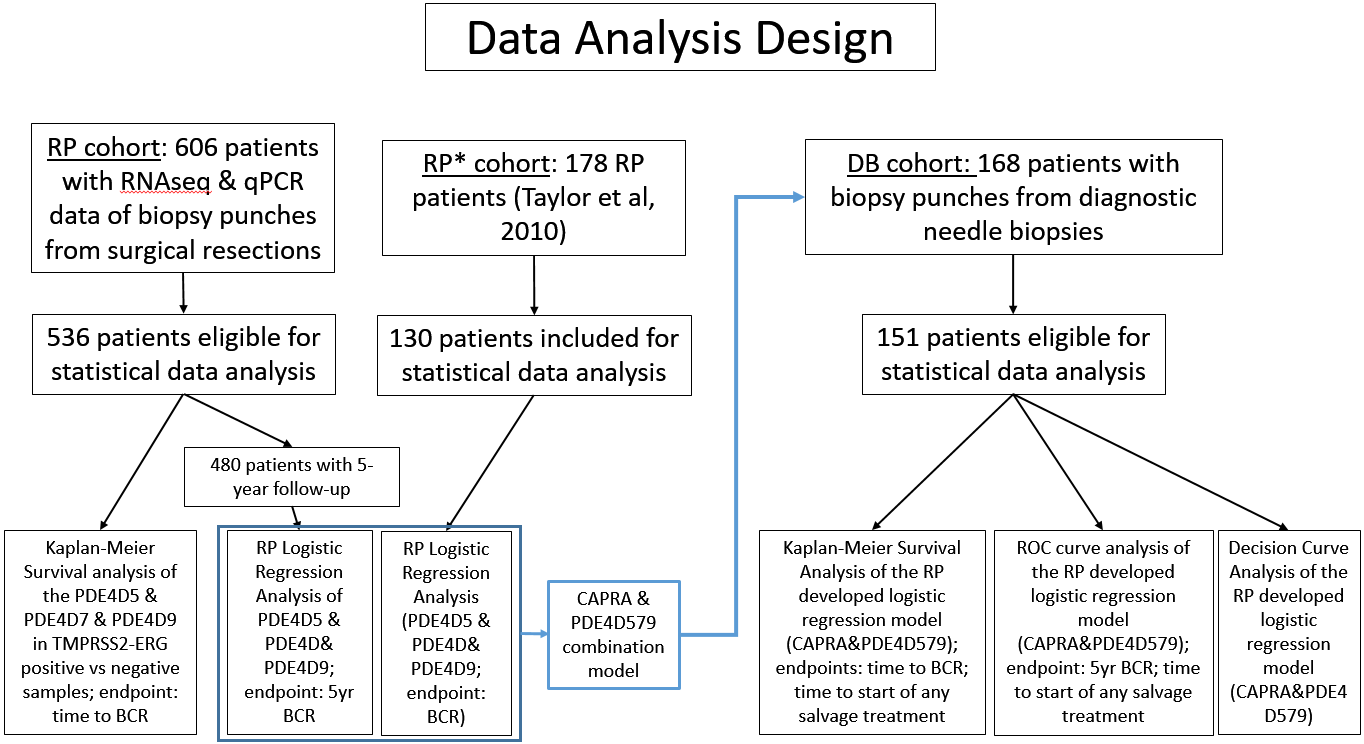


Analysis design of the generated PDE4D5/7/9 score data in the two patient study cohorts. The surgery cohort (RP) included 606 patients of which 536 were eligible for statistical data analysis after quality control of the RT-qPCR and the RNAseq data and after removal of patients with adjuvant and neo-adjuvant hormone therapy before or after surgery. The RP* cohort (Taylor et al, 2010) consisted of 178 patients of which 130 patients were included into this study. The diagnostic biopsy (DB) cohort comprised 168 patients of which 151 were eligible for statistical data analysis after quality control of the RT-qPCR data. For characteristics of the patient cohorts see Table 1 in the main manuscript.

**Supplementary Table 1**


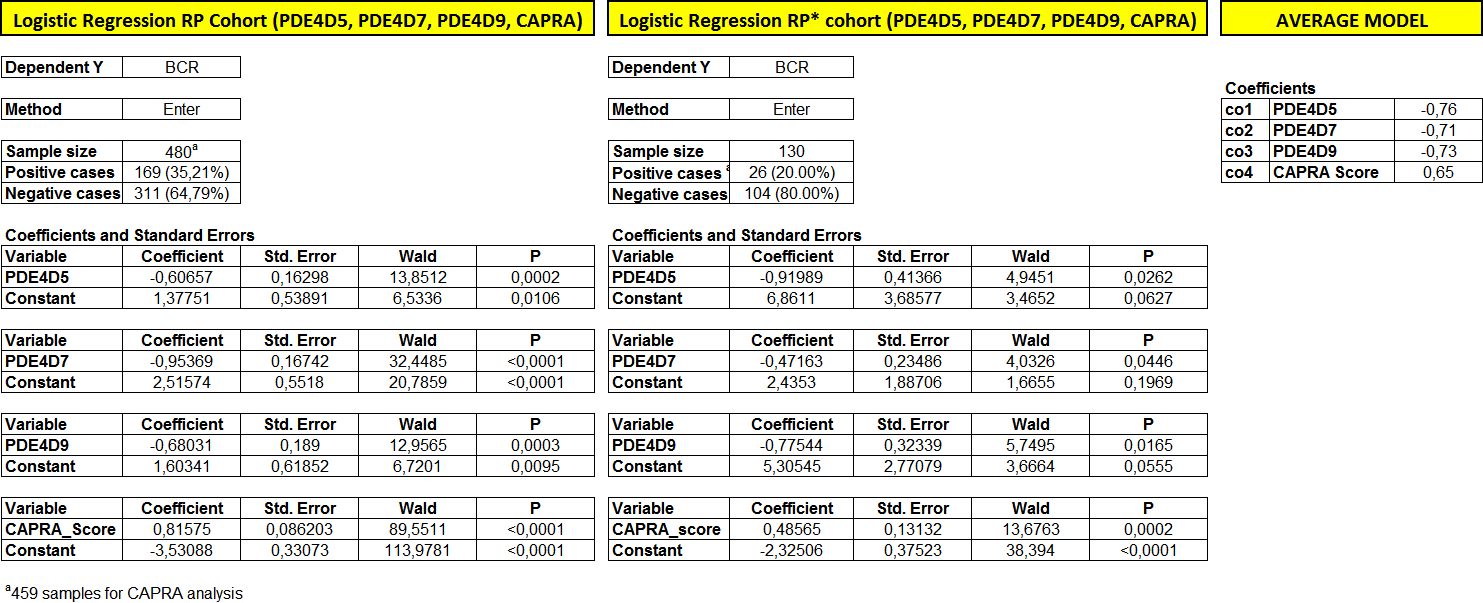


The CAPRA* & PDE4D579 score combination model was developed by logistic regression of the CAPRA score and the normalized PD4D5, PDE4D7, and PDE4D9 expression values in the RP patient cohort (n=480 with completed 5-years follow-up after surgery) and in the RP* cohort (n=130; Taylor et al, 2010). The logistic regression coefficients for the two cohorts were averaged to generate a mean coefficient for PD4D5, PDE4D7, and PDE4D9 and the CAPRA score based on the data of two independent patients cohorts in order to take the variation of different patient groups into account. These coefficients were used as weights in the final CAPRA & PDE4D579 regression model. Note: The CAPRA score is calculated based on Cooperberg et al, 2005; however, as the information on the number of positive biopsy cores was missing for the RP* cohort such that the CAPRA score for this cohort was calculated using only patient age, pre-operative PSA, biopsy Gleason score, and clinical stage. The influence of the missing information on the biopsy cores was very limited as tested on the RP as well as the DB cohort (data not shown).
